# The Human Vaginal Virome: Composition, Microbiome Interactions, and Implications for Bacterial Vaginosis, HPV Persistence, and Preterm Birth

**DOI:** 10.3390/microorganisms14092055

**Published:** 2026-09-15

**Authors:** José Eleutério Junior, Paulo César Giraldo, Renata Mírian Nunes Eleutério, Cristiana Rodrigues Teófilo

**Affiliations:** 1 Department of Women’s, Children’s and Adolescent Health, Faculty of Medicine, Federal University of Ceará, Fortaleza 60430-140, Brazil; 2 Department of Pathology, Federal University of Ceará, Fortaleza 60430-160, Brazil; crisrt@gmail.com; 3 Obstetrics and Gynecology Department, Faculty of Medicine, State University of Campinas, Campinas 13083-970, Brazil; paulocesargiraldo@gmail.com; 4 Prof. Eleutério Laboratory, Fortaleza 60110-371, Brazil; renatamneleuterio@gmail.com

**Keywords:** vaginal virome, vaginal microbiome, bacteriophages, vaginal dysbiosis, human papillomavirus, preterm birth, phage therapy

## Abstract

Important gaps remain in our understanding of the vaginal virome. Its composition, interactions with the bacterial microbiome and host immune system, and implications for vaginal homeostasis, human papillomavirus (HPV) persistence, and obstetric outcomes remain incompletely defined. The vaginal virome is a dynamic component of the female genital ecosystem. It includes viruses that infect human cells, bacteriophages, and endogenous viral elements. Observational studies have associated altered viral composition or diversity with vaginal dysbiosis, persistent HPV detection, and adverse pregnancy outcomes, including preterm birth. These relationships remain correlative, and causality has not been established. Bacteriophage-based therapies and recombinant endolysins may selectively target bacterial biofilms associated with bacterial vaginosis. However, most applications remain preclinical or in early clinical development. Better characterization of the interactions among viruses, bacteria, epithelial cells, and mucosal immunity may support future diagnostic biomarkers and precision therapeutic strategies in women’s health.

## 1. Introduction

Our understanding of female genital tract health has undergone a substantial transformation over the past two decades with the advent of next-generation sequencing (NGS) technologies, which have enabled detailed characterization of microbial communities associated with the human body [1,2]. In female reproductive health, these advances have demonstrated that vaginal homeostasis depends on dynamic interactions between the host, the local immune system, and a complex microbial ecosystem composed of bacteria, fungi, archaea, and viruses [3,4].

In most women of reproductive age, a healthy vaginal bacterial microbiome is characterized by the predominance of *Lactobacillus* species, particularly *L. crispatus*, *L. jensenii*, and *L. gasseri* [5,6,7]. These bacteria help maintain a vaginal pH below 4.5 through the production of lactic acid, bacteriocins, and other antimicrobial metabolites that inhibit the proliferation of potentially pathogenic microorganisms [6,7]. Alterations in vaginal microbial composition have been associated with bacterial vaginosis, reproductive tract infections, obstetric complications, and increased susceptibility to HPV infection [8,9,10,11].

In this review, eubiosis refers to a vaginal ecosystem compatible with mucosal homeostasis, generally characterized by ecological stability, preserved epithelial function, low inflammatory activity, and, in many reproductive-age populations, the predominance of lactic-acid-producing *Lactobacillus* species. Nevertheless, *Lactobacillus* dominance should not be considered a universal requirement for health across all individuals and populations. Dysbiosis refers to a disturbance in community composition or function associated with loss of ecological stability, frequently involving reduced abundance of protective lactobacilli, increased anaerobic diversity, higher vaginal pH, or increased inflammatory activity. Dysbiosis is not necessarily synonymous with symptoms or clinically diagnosed disease [3].

Bacterial community state types (CSTs) are compositional groupings used to classify vaginal bacterial communities according to their dominant taxa. CSTs I, II, III, and V are generally dominated by *Lactobacillus crispatus*, *L. gasseri*, *L. iners*, and *L. jensenii*, respectively, whereas CST IV comprises more diverse communities frequently enriched with anaerobic bacteria. CSTs are descriptive ecological categories rather than fixed biological or clinical states, and transitions between them may occur over time. Equivalent and reproducible vaginal viral community classifications have not yet been established [3,5,6].

Although the bacterial microbiome has been extensively investigated, the viral component of the vaginal ecosystem remains incompletely understood [4,12]. The term “virome” refers to the collection of viruses present within a specific biological niche, encompassing eukaryotic viruses, bacteriophages, and viral elements integrated within the genomes of resident microorganisms [13]. Recent metagenomic studies have demonstrated that the vaginal virome exhibits considerable diversity and may influence both the ecological stability of the vaginal microbiome and immune functions of the female genital mucosa [4,14].

Eukaryotic viruses identified in the female genital tract include members of the families *Papillomaviridae*, *Herpesviridae*, *Polyomaviridae*, and *Anelloviridae* [4,14,15]. Although some of these viruses are recognized for their clinical relevance, particularly HPV and herpesviruses, accumulating evidence suggests that certain components of the virome may also contribute to mucosal immune modulation and local homeostasis [4,16,17]. In parallel, bacteriophages, viruses that specifically infect and replicate within bacteria, represent a major component of the vaginal virome and can influence the bacterial community composition through bacterial lysis, horizontal gene transfer, and ecological remodeling [4,14].

In recent years, metagenomic and viromic studies have identified associations between alterations in the vaginal virome and clinically relevant conditions, including bacterial vaginosis, persistent HPV infection, cervical disease, and adverse pregnancy outcomes [4,14,15,17]. Although the causal nature of these associations remains uncertain, these observations support the hypothesis that the virome is a functionally relevant component of female reproductive health. Beyond its potential pathophysiological role, growing knowledge of bacteriophage biology has stimulated the development of innovative therapeutic approaches. Phage therapy and recombinant endolysins are being investigated as selective strategies for bacterial biofilms associated with bacterial vaginosis, particularly in the context of antimicrobial resistance [4].

Given the growing interest in the viral ecology of the female genital tract, a critical synthesis of the available evidence is warranted. Accordingly, this review examines vaginal virome composition, interactions with the bacterial microbiome and host immunity, and evidence relating specifically to bacterial vaginosis, HPV persistence and cervical disease, and preterm birth. These conditions represent the principal clinical areas for which evidence is currently available and do not encompass the entire spectrum of female reproductive health.

### Literature Search Strategy

A structured narrative literature search was conducted in PubMed/MEDLINE, Scopus, Web of Science, and Embase from database inception through August 2026. Search terms combined concepts related to the anatomical site (vaginal, cervicovaginal, or female genital tract) with virome-related terms (virome, virus, viral metagenomics, bacteriophage, phage, or prophage). Additional searches incorporated condition-specific terms, including bacterial vaginosis, dysbiosis, human papillomavirus, HPV persistence, cervical disease, pregnancy, preterm birth, phage therapy, and endolysin. Reference lists of relevant articles were screened to identify additional publications, and trial registries were consulted to determine the translational status of investigational phage-derived products.

Eligible sources included human observational studies, longitudinal cohorts, metagenomic and viromic investigations, experimental and ex vivo studies relevant to biological mechanisms, clinical trials, and authoritative reviews addressing the vaginal virome or directly related cervicovaginal ecological processes. Studies confined to non-genital anatomical sites, reports without relevance to vaginal viral ecology, non-informative conference abstracts, and duplicate publications were excluded. Priority was given to recent primary studies, while older publications were retained when they provided foundational definitions, methods, or biological concepts. Evidence was synthesized narratively because of substantial heterogeneity in sampling, sequencing, bioinformatic pipelines, populations, and reported outcomes. No formal meta-analysis or risk-of-bias assessment was performed.

## 2. Composition and Ecology of the Vaginal Virome

The vaginal virome is a complex and dynamic component of the microbial ecosystem of the female genital tract. Although it has historically received less attention than the bacterial microbiome, metagenomic studies indicate that viral communities are closely integrated with mucosal ecology and the vaginal bacterial microbiome [4,12,14].

Vaginal virome composition varies considerably among individuals and is influenced by age, sexual activity, hormonal status, contraceptive use, pregnancy, bacterial community structure, and vaginal dysbiosis. These factors, together with differences in sampling and analytical methods, complicate the definition of a stable core vaginal virome [4,14].

Bacteriophages frequently dominate vaginal viral sequence datasets and are closely linked to the relatively low-complexity bacterial communities that often characterize *Lactobacillus*-dominated community state types (CSTs). The eukaryotic fraction includes both asymptomatic viral carriage and clinically relevant infections, although prevalence estimates vary substantially according to the population, specimen type, nucleic-acid preparation, and sequencing platform [4,14].

Rather than indicating infection or disease, the detection of viral sequences in the vaginal mucosa can occur in healthy asymptomatic women. Human-Microbiome-Project-based analyses have identified several eukaryotic viral families in such populations, with alphapapillomaviruses among the most frequently detected [14].

Interactions between viral and bacterial communities are likely to contribute to vaginal ecological stability. Under eubiotic conditions, *Lactobacillus*-dominated bacterial communities coexist with their associated bacteriophages within an environment characterized by low inflammation, preserved epithelial integrity, and appropriate local immune function [14].

Recent evidence indicates that cervicovaginal virome composition is associated with bacterial community structure and genital inflammation. These observations do not establish that viral communities initiate transitions between eubiosis and dysbiosis; virome alterations may instead contribute to, amplify, result from, or simply reflect ecological and inflammatory disturbances [14,17].

Taken together, current evidence supports the concept that the vaginal virome is not merely a collection of resident viral particles but rather a functionally integrated component of the female genital ecosystem that may influence both microbial ecology and host immune responses.

## 3. Eukaryotic Viruses and Mucosal Immunity

The eukaryotic component of the genital virome comprises viruses capable of infecting human cells and includes asymptomatic, persistent, latent, transient, and clinically pathogenic viruses. Frequently detected or clinically relevant families include *Papillomaviridae*, *Anelloviridae*, *Herpesviridae*, *Polyomaviridae*, *Adenoviridae*, and *Poxviridae*. *Molluscum contagiosum virus*, a member of *Poxviridae*, primarily causes lesions of the skin and may involve the anogenital region. Its relevance illustrates that the broader genital viral ecology extends beyond viruses consistently recovered from vaginal metagenomic samples. Accordingly, detection in the vagina, clinical involvement of adjacent genital tissues, and stable membership of the vaginal virome should not be treated as equivalent concepts [18].

Viruses frequently identified in asymptomatic women include members of the families *Anelloviridae*, *Polyomaviridae*, and *Papillomaviridae* [14,15]. Anelloviruses, particularly torque teno viruses (TTVs), have attracted increasing attention because of their high prevalence in healthy individuals and their association with host immune status [16,19]. Although definitive evidence for either a protective or pathogenic role is lacking, anellovirus abundance has been proposed as an indirect marker of immune status in several clinical settings [16].

The vaginal mucosa is an immunological interface in which epithelial and immune cells recognize viral nucleic acids and proteins through endosomal and cytosolic pattern-recognition receptors. These pathways can activate interferon-regulatory and nuclear factor-κB signaling, influencing type I and type III interferons, inflammatory cytokines, immune-cell recruitment, and epithelial-barrier function. In parallel, phage-mediated changes in bacterial abundance or bacterial lysis may alter exposure to bacterial products and thereby modify Toll-like-receptor and other innate immune pathways [20,21].

Observational studies have associated alterations in cervicovaginal virome composition, including differential abundances of anelloviruses, with genital inflammation and altered cytokine profiles [17,21]. These findings support a relationship between the viral ecosystem and mucosal immune state, although the direction and causal significance of these associations remain uncertain.

Collectively, these observations support investigation of the vaginal virome as a potential modulator or marker of mucosal immune states. Most available findings remain observational, and detection of a viral sequence does not necessarily demonstrate active replication, biological activity, or causal involvement in disease [16,17,21].

## 4. Bacteriophages and Regulation of the Vaginal Microbiome

Bacteriophages constitute a major component of the vaginal virome and can shape bacterial communities through lysis, horizontal gene transfer, and selective pressure. Their replication strategies differ: virulent phages follow a lytic developmental program in which phage replication culminates in bacterial lysis and release of progeny virions, whereas temperate phages can establish lysogeny. During lysogeny, the phage genome is maintained within the bacterial host, commonly as an integrated prophage, without immediate lysis. Strictly lytic phages do not undergo lysogeny [22,23,24].

Environmental or cellular stress may induce a temperate prophage to enter lytic replication. This transition is influenced by bacterial physiology, multiplicity of infection, extracellular signals, and phage regulatory circuits. Integration is not obligatory in every lysogenic state because some temperate phage genomes may be maintained extrachromosomally [23,24].

In eubiotic vaginal ecosystems, bacteriophages and *Lactobacillus* species appear to coexist within a dynamic ecological equilibrium that may contribute to microbial resilience [14,25]. In addition, bacteriophages can facilitate horizontal gene transfer and influence bacterial adaptation and fitness [22,23,24].

**Figure 1:**
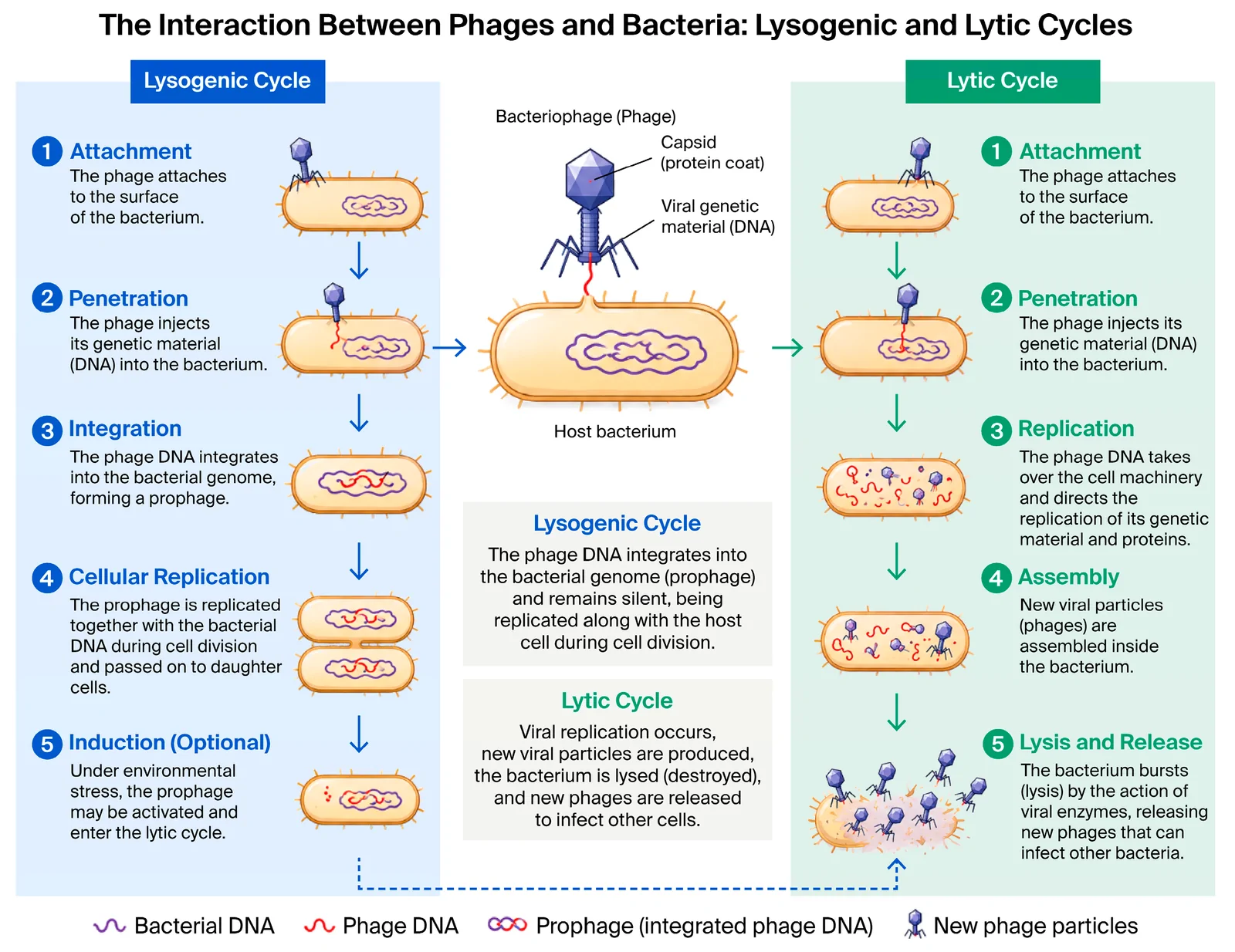
Lytic and lysogenic bacteriophage life cycles and their potential effects on vaginal bacterial populations. Virulent phages follow a lytic program in which infection is followed by genome replication, assembly of progeny virions, bacterial-cell lysis, and release of new phage particles. Temperate phages can establish lysogeny, during which the phage genome is maintained in the bacterial host, commonly as an integrated prophage, and replicated with the bacterial chromosome. Environmental or cellular stress may induce some prophages to enter lytic replication. Strictly lytic phages do not undergo lysogeny. In the vaginal ecosystem, bacterial lysis may alter bacterial abundance, release microbial products, and influence community structure.

**Figure 2:**
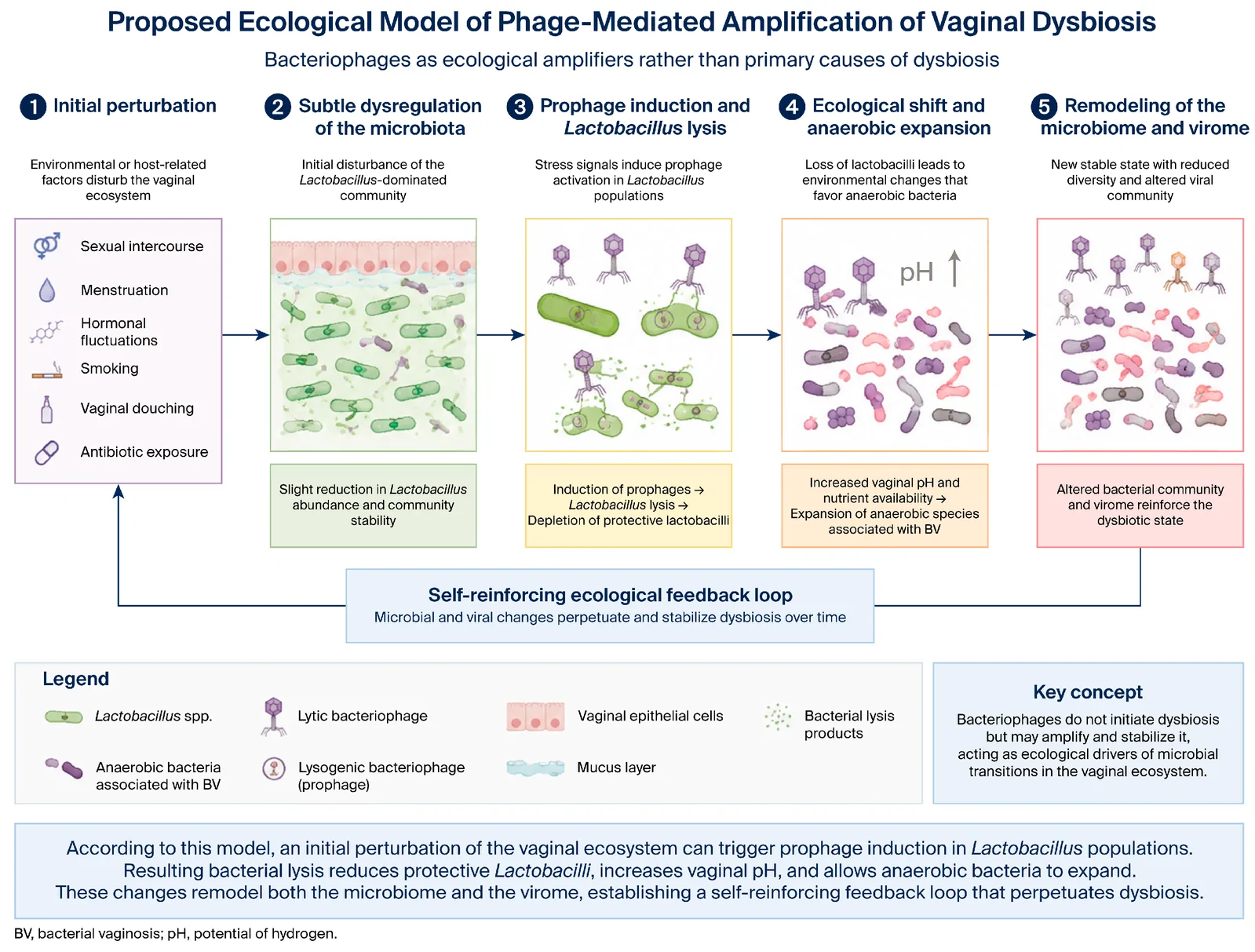
Hypothetical model of phage-mediated amplification of vaginal dysbiosis. Host-related or environmental perturbations may destabilize a previously resilient vaginal bacterial community. In susceptible *Lactobacillus* populations, cellular stress may induce resident temperate prophages, leading to bacterial lysis and further depletion of protective lactobacilli. Increased vaginal pH and altered nutrient availability may then facilitate expansion of bacterial-vaginosis-associated anaerobes. These changes may remodel the phage community and reinforce ecological instability. The model proposes that bacteriophages may amplify or perpetuate dysbiosis after an initial disturbance; it does not establish bacteriophages as primary causes of bacterial vaginosis.

Current studies demonstrate associations between bacteriophage composition and vaginal bacterial community structure but do not establish phages as independent initiators of dysbiosis. A more conservative model is that an initial host, behavioral, microbial, or environmental perturbation destabilizes a *Lactobacillus*-dominated community; prophage induction or lytic activity may then amplify the disturbance by accelerating depletion of susceptible bacteria, releasing bacterial products, or altering competitive relationships (Figure 1). Increased vaginal pH and nutrient availability may facilitate anaerobic expansion, while reciprocal remodeling of the bacterial microbiome and virome may perpetuate ecological instability. In this hypothesis-generating model, phages act as potential ecological amplifiers rather than proven primary causes of bacterial vaginosis (Figure 2) [14,17,25].

## 5. Vaginal Virome and Dysbiosis: Current Evidence

Bacterial vaginosis is the most common form of vaginal dysbiosis among women of reproductive age and is characterized by the replacement of *Lactobacillus*-dominated communities with polymicrobial communities enriched with anaerobic bacteria [8]. Despite substantial advances in understanding this condition, the mechanisms underlying its onset and recurrence remain incompletely understood.

Recent studies suggest that alterations in the vaginal virome are associated with ecological instability accompanying vaginal dysbiosis [14,17,25]. Compared with *Lactobacillus*-dominated CSTs, bacterial vaginosis is associated with changes in viral diversity, bacteriophage abundance, and the viral community structure [14,17,25]. The composition of the vaginal virome appears to be closely linked to the bacterial microbiome, particularly the relative abundance of protective lactobacilli and bacterial vaginosis-associated anaerobes. Larger longitudinal studies are needed to determine whether reproducible viral community state types can be defined and whether they consistently parallel bacterial community state types [14,17].

Beyond quantitative differences, researchers have identified viral genes associated with integration, recombination, and bacterial adaptation in vaginal communities exhibiting dysbiosis. These observations are compatible with a role for viruses in ecological remodeling but do not establish whether viral changes precede or follow bacterial dysbiosis [17,23,24].

The vaginal virome should therefore be considered within a dynamic bacteria–virus–immunity–epithelium network. At present, its components are best interpreted as potential contributors, ecological amplifiers, or biomarkers of dysbiosis rather than established independent causes [4,14,17] (Table 1).

## 6. Vaginal Virome, HPV Persistence, and Cervical Carcinogenesis

Persistent infection with oncogenic human papillomavirus (HPV) genotypes, particularly HPV16 and HPV18, is the principal etiological driver of high-grade squamous intraepithelial lesions (HSILs) and cervical cancer. However, only a minority of HPV infections persist and progress, indicating that additional host, microbial, and environmental factors influence disease outcomes [11,21,26,27].

Accumulating evidence indicates that vaginal bacterial community composition is associated with the natural history of HPV infection. *Lactobacillus crispatus*-dominated microbiota are generally associated with more favorable HPV outcomes, whereas diverse anaerobe-rich communities are associated with persistent HPV detection and cervical precancer. The relationship is likely bidirectional because HPV-infected epithelial cells may themselves alter innate immune pathways, epithelial differentiation, antimicrobial peptides, and the local metabolic environment [11,26,27,28,29,30].

**Figure 3:**
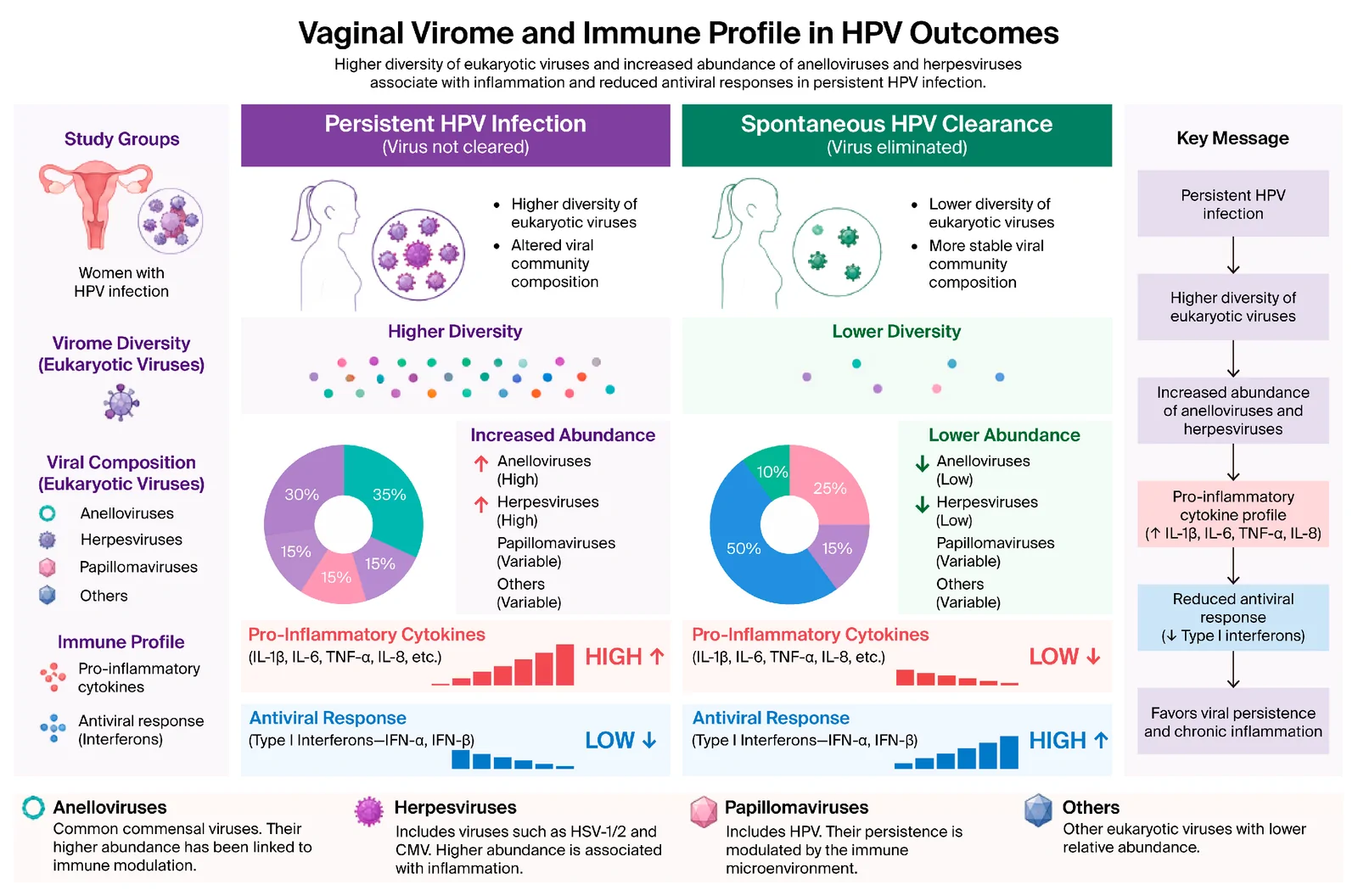
Conceptual relationship between vaginal virome composition, mucosal immune profiles, and HPV outcomes. Greater eukaryotic viral diversity and altered abundance of anelloviruses or herpesviruses have been reported in association with inflammatory profiles and persistent HPV detection. These findings are observational and do not establish a unidirectional pathway. Viral and bacterial components may be sensed by epithelial and immune-cell pattern-recognition receptors, influencing interferon signaling, inflammatory cytokines, immune-cell recruitment, and epithelial-barrier integrity. These interacting processes may affect, or result from, the local environment associated with HPV control. Percentages shown are illustrative and should not be interpreted as pooled quantitative estimates. An upward arrow represents high, and a downward arrow represents low. Sources: Audirac-Chalifour et al. [21]; Bautista et al. [27].

More recently, the vaginal virome has emerged as another potential modulator of this process. An observational study reported greater eukaryotic viral diversity and altered viral community composition across cervical disease states [15]. Higher abundances of anelloviruses and herpesviruses have also been linked to altered immune and inflammatory profiles, although these relationships are not yet sufficient to establish causality [16,17,21,31] (Figure 3).

A recent study of the cervicovaginal DNA virome showed that non-papillomavirus viruses, including anelloviruses and bacteriophages, were associated with genital inflammation and bacterial community composition. HPV abundance and contig richness were not the principal correlates of these microenvironmental features; instead, virome alterations included anellovirus expansion and bacteriophage–bacterium associations involving bacterial vaginosis-associated taxa [17]. These findings support the view that the cervicovaginal virome may serve as a dynamic indicator of local ecological and inflammatory states rather than a direct marker of HPV infection alone.

Taken together, current evidence supports a model in which bacterial dysbiosis, altered virome composition, epithelial changes, and mucosal inflammation coexist within a microenvironment associated with persistent HPV detection. The vaginal virome is more plausibly viewed as one component of this ecological network than as an independent determinant of HPV persistence or cervical carcinogenesis (Table 1). Because the evidence is predominantly observational, it remains uncertain whether virome alterations contribute to HPV persistence, result from HPV-associated epithelial and immune changes, or reflect shared ecological disturbances [11,17,26,27,28,29].

## 7. Vaginal Virome and Preterm Birth

Spontaneous preterm birth remains a leading cause of neonatal morbidity and mortality worldwide. Genetic, environmental, immunological, and infectious factors contribute to its etiology, and accumulating evidence indicates that the vaginal microbiome during pregnancy is associated with adverse obstetric outcomes [32,33,34,35].

During uncomplicated pregnancies, vaginal bacterial communities often become more *Lactobacillus*-dominant and less diverse, a pattern associated with reproductive tract stability [35,36]. Longitudinal analysis of the vaginal eukaryotic DNA virome has shown that viral communities also vary during pregnancy and that greater viral richness can be associated with preterm birth [37].

Alterations in the vaginal virome during pregnancy have been associated with preterm birth, although no single virus has been established as a causal agent [37]. The underlying mechanisms remain incompletely understood, and interactions between viruses, the bacterial microbiome, and host immunity may contribute indirectly to gestational inflammatory states.

Several non-mutually exclusive mechanisms may explain these associations. Altered bacteriophage dynamics could contribute to the depletion of protective *Lactobacillus* populations and the expansion of inflammation-associated anaerobic communities [25]. In addition, phage-mediated bacterial lysis can release bacterial products that stimulate innate immune pathways; excessive inflammatory signaling is a recognized component of pathways leading to cervical remodeling and preterm parturition [22,25,34]. Importantly, there is currently no evidence that vaginal bacteriophages directly infect or damage the amnion or chorion. Their proposed contribution is therefore indirect and mediated through effects on the vaginal microbial ecosystem and host inflammatory responses.

Overall, available evidence remains insufficient to establish a causal relationship between vaginal virome alterations and spontaneous preterm birth. Longitudinal studies integrating virome, microbiome, metabolome, and host immune profiling will be necessary to determine whether viral alterations have independent prognostic value or instead represent biomarkers of ecosystem instability during pregnancy [38].

Taken together, these observations support the concept that the vaginal virome may influence obstetric outcomes by modulating the ecological and immunological stability of the cervicovaginal environment rather than by directly interacting with gestational tissues [25,32,33,34,35,36,37] (Table 1).

## 8. Phage Therapy and Recombinant Endolysins

Advances in vaginal viral ecology have stimulated interest in bacteriophages and phage-derived proteins as therapeutic strategies. The level of supporting evidence varies substantially and must be distinguished from established clinical practice. Conventional metronidazole- or clindamycin-based regimens remain guideline-supported treatments for bacterial vaginosis, whereas whole-phage therapy remains experimental and endolysin-based interventions are in early translational development [19,25,39,40,41,42,43].

Classical phage therapy uses intact bacteriophages to infect susceptible bacterial hosts, whereas endolysin therapy uses purified phage-derived enzymes that cleave bacterial peptidoglycan without requiring viral replication. Both approaches may offer greater target specificity than broad-spectrum antibiotics. For bacterial vaginosis, however, whole-phage therapy remains predominantly at the discovery and preclinical stages [19,40,41,42,44].

Whole-phage interventions face challenges arising from the polymicrobial architecture of bacterial-vaginosis biofilms, narrow host range, phage resistance, uncertain biofilm penetration, formulation stability, possible immune neutralization, and the potential need for individualized phage combinations. Replicating products also raise genomic-safety and environmental-release considerations [19,25,42,43,45].

PM-477, an engineered endolysin derived from *Gardnerella* prophages, has demonstrated selective activity against planktonic cells and biofilms in vitro and in ex vivo vaginal samples while largely preserving lactobacilli (Table 1). These findings remain preclinical or ex vivo and do not establish clinical efficacy. Translational development has progressed to BNT331, an optimized recombinant endolysin-based investigational product derived from the PM-477 program. BNT331 has completed a first-in-human phase 1 randomized, double-blind, placebo-controlled study involving healthy women and women diagnosed with bacterial vaginosis (BNT331-01; NCT06469164) [39,40,41,46].

An updated search of ClinicalTrials.gov identified one registered interventional study directly evaluating a phage-derived product for bacterial vaginosis: the phase 1 BNT331-01 trial (NCT06469164), which is listed as completed. We identified no registered human trial of PM-477 itself or whole-phage therapy for bacterial vaginosis. Table 2 summarizes the registered endpoints and current development gaps [46].

Larger controlled trials are required to establish the efficacy, recurrence prevention, optimal dosing, long-term safety, and comparative effectiveness of BNT331 and other phage-derived interventions. Regulatory implementation will also require standardized manufacturing, characterization of identity and potency, removal of bacterial contaminants, formulation stability, evaluation of local and systemic immunogenicity, and assessment of unintended effects on commensal organisms. Regulatory pathways for replicating or personalized phage products remain heterogeneous internationally [47]. Neither whole-phage nor recombinant endolysin therapy should currently be considered an established replacement for guideline-recommended treatment of bacterial vaginosis (Table 3).

## 9. Limitations of Current Knowledge

Despite the rapid expansion of vaginal virome research, important methodological and conceptual limitations continue to hinder progress. One major technical challenge is the low microbial and viral biomass of vaginal specimens, which increases susceptibility to contamination during sample collection, nucleic acid extraction, library preparation, and sequencing [48].

Another major limitation is the absence of standardized methodologies for virome characterization. Differences in sample processing, sequencing platforms, genome assembly, taxonomic annotation, and bioinformatic workflows complicate comparisons across studies and reduce reproducibility [49,50,51,52,53].

Furthermore, most available evidence is derived from cross-sectional observational studies, which preclude causal inference. Consequently, it remains uncertain whether virome alterations precede microbial dysbiosis, arise as a consequence of ecological disruption, or simply reflect ongoing inflammatory processes [4,14,17,37].

An additional challenge is the large proportion of viral sequences that remain taxonomically and functionally uncharacterized—a phenomenon commonly referred to as “viral dark matter.” Incomplete viral reference databases limit confident taxonomic and functional assignment, potentially leading to underestimation of viral diversity and obscuring biologically relevant virus–host interactions [49,51].

Another important limitation is the reliance on taxonomic descriptions rather than functional characterization. Metagenomic sequencing can identify viral genomes but provides limited information regarding viral activity, replication states, bacteriophage life cycles, or the biological consequences of virus–host interactions. The integration of metagenomics with metatranscriptomics, metaproteomics, metabolomics, and experimental validation will be essential to establish functional relevance [49,51,53] (Table 4).

Finally, distinguishing resident members of the vaginal virome from transient viruses introduced through sexual activity, environmental exposure, or contamination is a major challenge. Without longitudinal sampling, defining a stable core vaginal virome remains difficult [4,12,14,48].

### Causality Challenges

Establishing causality is a central challenge in vaginal virome research. Most available studies are cross-sectional or observational and can identify associations but cannot determine whether virome alterations precede, contribute to, or result from vaginal dysbiosis, HPV persistence, or adverse obstetric outcomes. Reverse causation is plausible because inflammation, epithelial disruption, antimicrobial exposure, hormonal changes, sexual activity, and bacterial-community shifts may themselves modify viral abundance and detectability. Residual confounding and temporal variability of the cervicovaginal ecosystem further complicate causal interpretation [4,14,17,37,48,49,50,51,52,53,54].

Detection of a viral sequence does not necessarily indicate active replication or biological activity. Viral nucleic acids may originate from actively replicating viruses, latent or integrated elements, extracellular particles, lysed bacterial cells, transient exposure, or contamination. Relative abundance can also change because of variation in other community members without a corresponding change in the absolute abundance of the virus of interest [12,13,14,49,51,53].

Moving from association to causation will require prospective longitudinal cohorts with repeated sampling before and after outcomes; measurement of absolute viral and bacterial abundance; strain-resolved virus–host linkage; integration of metagenomic, metatranscriptomic, metabolomic, and mucosal immune data; mechanistic experimental models; and controlled interventions testing whether modification of a defined viral component changes a clinically relevant outcome. Until such evidence becomes available, vaginal virome alterations should be interpreted as potential contributors, ecological amplifiers, consequences, or biomarkers of ecosystem disruption rather than established independent causes of disease [48,49,50,51,52,53,54].

## 10. Future Perspectives

Advances in metagenomics, metatranscriptomics, and long-read or single-molecule sequencing are expected to expand understanding of viral ecology in the female genital tract. Long reads can improve recovery of complete viral genomes, structural variants, prophage context, and virus–host linkage, while hybrid sequencing can combine these advantages with the base accuracy and throughput of short reads [50,53,54,55]. A major priority will be integration of metagenomics with metatranscriptomics, metaproteomics, metabolomics, and host immune profiling to characterize viral activity and functional interactions rather than composition alone.

Beyond naturally occurring bacteriophages, advances in synthetic biology are creating opportunities to engineer phages with altered host ranges, enhanced activity against biofilms, or the capacity to deliver genetic payloads that modify bacterial phenotypes. Although these approaches remain largely experimental, they illustrate the broader potential of phage-based technologies for precision microbiome engineering [19,42,45].

An emerging frontier is functional viromics, which seeks to determine how viral communities influence microbial ecology and host physiology rather than simply cataloging viral genomes. Combining experimental models with high-resolution multi-omics technologies will be critical for identifying the biological mechanisms through which bacteriophages and eukaryotic viruses shape vaginal homeostasis and disease [49,51,53].

## 11. Conclusions

Rather than an isolated collection of resident viruses, the vaginal virome is an integral and dynamic component of the microbial ecosystem of the female genital tract. Available evidence indicates complex associations among viral communities, the bacterial microbiome, epithelial function, and host immunity in bacterial vaginosis, HPV persistence and cervical disease, and preterm birth. These selected conditions represent the best-developed areas of current investigation but do not exhaust the potential implications of the genital virome for female reproductive health.

Current knowledge remains largely observational, and virome alterations cannot yet be assigned an independent causal role in dysbiosis, persistent HPV detection, or adverse obstetric outcomes. Similarly, whole-phage therapy remains experimental, while recombinant endolysins have progressed from preclinical and ex vivo evaluation to early clinical development without yet establishing efficacy or a place in routine care.

Establishing the clinical relevance of the vaginal virome will require robust longitudinal studies, standardized metagenomic and bioinformatic methods, functional experimental validation, and the integration of multi-omics approaches. Such advances may facilitate the translation of virome-based knowledge into diagnostic, prognostic, and therapeutic strategies in female reproductive health.

## Figures and Tables

**Table 1:** Summary of current evidence linking the vaginal virome to selected clinical conditions.

Clinical Condition	Reported Virome Changes	Main Viral Components	Type of Evidence	Potential Clinical Significance
Bacterial vaginosis	Altered viral diversity and phage composition associated with anaerobe-rich, *Lactobacillus*-depleted communities	Phages associated with lactobacilli and BV-associated anaerobes; non-HPV eukaryotic viruses in some datasets	Predominantly cross-sectional metagenomic or viromic studies	Possible marker or amplifier of ecological instability; causality and diagnostic utility remain unproven
HPV persistence and cervical disease	Greater eukaryotic viral diversity and altered viral composition reported across HPV outcome or cervical disease groups	Papillomaviruses, anelloviruses, herpesviruses, and bacteriophages	Cross-sectional and limited longitudinal observational evidence	May reflect or modify the local immune–microbial environment; no independent causal role established
Preterm birth	Greater viral richness or altered eukaryotic DNA virome composition in some pregnancy cohorts	Eukaryotic DNA viruses and bacteriophages; no single causal virus identified	Limited observational longitudinal evidence	Potential biomarker of ecosystem instability; predictive value and causality require validation
BV treatment	Selective targeting of *Gardnerella* or associated biofilms with limited disruption of lactobacilli	Whole phages and engineered recombinant endolysins, including PM-477-derived BNT331	Whole phages: experimental; PM-477: in vitro/ex vivo; BNT331: completed phase 1	Potential precision therapy; clinical efficacy, recurrence prevention, long-term safety, and approval remain unestablished

**Table 2:** Registered clinical development of phage-derived interventions for bacterial vaginosis (ClinicalTrials.gov search updated 11 September 2026).

Product or Modality	Registry Identifier and Status	Phase, Design, and Population	Primary Endpoints	Secondary Endpoints or Evidence Gap
BNT331; optimized recombinant endolysin derived from the PM-477 program	NCT06469164; completed; no registry results posted as of 11 September 2026	Phase 1; randomized, double-blind, placebo-controlled; 102 adult premenopausal women: healthy participants in Part A and participants with BV in Part B	Treatment-emergent adverse events and serious adverse events: through 7 days after a single dose in Part A and through 120 days after the first dose in Part B	Pharmacokinetics; anti-drug antibodies; clinical cure; Nugent score < 4; composite clinical and microbiological response
PM-477; engineered Gardnerella-specific recombinant endolysin	No registered human interventional trial identified	Preclinical and ex vivo development	No clinical endpoint registered	Published evidence addresses selective killing of Gardnerella and disruption of BV-associated biofilms; human efficacy and safety remain unestablished
Whole-bacteriophage therapy for BV	No registered BV-specific human interventional trial identified	Discovery and preclinical stage	No clinical endpoint registered	Clinical development requires defined host range, formulation, dosing, biofilm activity, genomic safety, resistance monitoring, and regulatory standardization

**Table 3:** Conventional and phage-derived therapeutic approaches for bacterial vaginosis.

Approach	Mechanism	Current Evidence	Potential Advantages	Principal Limitations
Metronidazole or clindamycin	Inhibition or killing of susceptible anaerobic bacteria	Established clinical evidence and guideline recommendations	Available formulations; familiar dosing; short-term efficacy	Frequent recurrence; incomplete biofilm disruption; adverse effects; resistance; ecological effects
Whole-bacteriophage therapy	Replicating phages selectively infect and lyse susceptible hosts	Experimental and preclinical for BV; no established clinical efficacy	High specificity; self-amplification with susceptible hosts; possible activity against resistant bacteria	Narrow host range; polymicrobial BV; resistance; biofilm penetration; formulation; genomic safety; immunogenicity; regulation
Recombinant endolysins	Purified phage-derived enzymes cleave bacterial peptidoglycan	PM-477: in vitro and ex vivo; BNT331: completed first-in-human phase 1	Rapid lysis; potential Gardnerella specificity; preservation of lactobacilli; non-replicating product	Early clinical development; efficacy and recurrence prevention not established; delivery, dosing, stability, immunogenicity, and manufacturing requirements
Combination strategies	Phages or endolysins combined with antibiotics, biofilm disruptors, or microbiome restoration	Conceptual or preclinical	Potential complementary activity against biofilms and polymicrobial communities	Optimal combinations, treatment sequence, ecological effects, safety, and benefit remain undetermined

**Table 4:** Comparison of sequencing approaches applicable to vaginal virome research.

Approach	Principal Information	Main Strengths	Main Limitations	Most Appropriate Applications
Short-read shotgun DNA metagenomics	DNA viral sequences from total-community or virus-enriched preparations	High base accuracy; high throughput; established pipelines; comparatively lower cost	Fragmented assemblies; limited resolution of repeats and structural variation; uncertain host assignment; underrepresentation of RNA viruses	Comparative profiling of DNA viral communities and detection of known taxa
Long-read DNA sequencing	Long or near-complete DNA molecules and viral genomes	Improved genome continuity; structural variation, prophage, genomic-context, and virus–host resolution	Higher nucleic-acid input; platform-dependent errors; cost; difficult application to low-biomass samples	Genome-resolved viromics, prophage characterization, strain analysis, and virus–host linkage
Metatranscriptomics	Viral and host RNA transcripts, including RNA viruses	Detection of RNA viruses; transcriptional activity; host and microbial functional responses	RNA instability; host/rRNA background; extraction and depletion bias; transcription does not prove productive replication	RNA virome characterization and functional assessment
Hybrid and integrated multi-omics	Combined short/long reads with transcriptomic, proteomic, metabolomic, or immune data	Greater completeness; improved functional interpretation and virus–host linkage	Higher cost and computational complexity; demanding processing; batch effects; need for standardized integration	Mechanistic studies, biomarker development, and longitudinal ecosystem analysis

## Data Availability

No new data were created or analyzed in this study. Data sharing is not applicable to this article.

## Abbreviations

The following abbreviations are used in this manuscript:

BV	bacterial vaginosis
CMV	cytomegalovirus
CST	community state type
EBV	Epstein–Barr virus
HPV	human papillomavirus
HSIL	high-grade squamous intraepithelial lesion
MCV	molluscum contagiosum virus
HSV	herpes simplex virus
NGS	next-generation sequencing
PAMP	pathogen-associated molecular pattern
PRR	pattern recognition receptor
TLR	Toll-like receptor
TTV	torque teno virus
